# Cephaeline promotes ferroptosis in breast cancer via p53/SLC7A11/GPX4 axis

**DOI:** 10.1007/s40199-026-00619-9

**Published:** 2026-06-19

**Authors:** Xiaohong Li, Jinghan Yu, Beibei Lin, Yudiao Gong

**Affiliations:** 1 The People’s Hospital of Cangnan, The Affiliated Cangnan Hospital, Wenzhou Medical University, Wenzhou, China; 2 The Sixth Affiliated Hospital of Wenzhou Medical University, Lishui People’s Hospital, Wenzhou Medical University, Lishui, China

**Keywords:** Breast cancer, Cephaeline, Ferroptosis, P53, SLC7A11

## Abstract

**Background:**

Cephaeline (CPL), a bioactive compound derived from the medicinal plant Ipecacuanha, has demonstrated inhibitory effects on several tumor types. Nevertheless, its role in breast cancer and the underlying molecular mechanisms remain largely unexplored.

**Objectives:**

This research aimed to investigate the anti-cancer potential of CPL in breast cancer cells.

**Methods:**

The IC50 of CPL was determined using the CCK-8 assay. Subsequently, the effects of CPL on cell migration and proliferation were assessed through wound healing (scratch) and colony formation assays, respectively. Additionally, MDA and GSH levels were quantified using ELISA, while ROS production was visualized via DHE staining. Intracellular iron content was measured using an iron assay kit. Furthermore, the expression levels of p53, SLC7A11, and GPX4 were evaluated using Western blot. All experiments were performed in triplicate and independently repeated at least three times, and data were analyzed using Student’s t-test or one-way ANOVA, as appropriate.

**Results:**

CPL significantly inhibited the proliferation and viability of 4T1 and MDA-MB-231 breast cancer cells in a dose-dependent manner, with IC50 values of 38.89 nM and 50.29 nM, respectively, and concomitantly suppressed cell migration and colony formation at higher concentrations. It also increased intracellular MDA and ROS and downregulated GSH, SLC7A11 and GPX4, inducing ferroptosis. siRNA knockdown of p53 attenuated CPL's effects, indicating p53's key role in CPL's anti-cancer activity.

**Conclusions:**

This study provides evidence that CPL exerts anti-breast cancer effects by promoting ferroptosis through the p53/SLC7A11/GPX4 axis, highlighting its therapeutic potential as a novel agent for cancer treatment.

## Background

Breast cancer, the most prevalent female malignancy globally [1], remains a significant public -health concern [2], accounting for about 30% of all female cancer cases and 12% of cancer-related deaths [3]. Current clinical treatment mainly involves surgery, radiotherapy, and chemotherapy, with recent progress in molecularly targeted and immunotherapies expanding treatment options [4]. However, tumor cell drug resistance still hampers treatment effectiveness, highlighting the need to discover new cell death mechanisms to break through therapeutic bottlenecks.

Ferroptosis is a distinct form of regulated cell death characterized by iron-dependent lipid peroxidation and redox imbalance [5, 6]. Its hallmarks include GPX4 inactivation, disruption of antioxidant defenses, and accumulation of lipid peroxidation products, ultimately leading to oxidative cell death [7, 8]. At the molecular level, it's driven by reactive oxygen species (ROS) surge due to dysregulated iron metabolism and a regulatory network distinct from classical cell death pathways [9, 10]. Importantly, therapeutic targeting of ferroptosis has emerged as a promising strategy to suppress tumor growth and overcome resistance to conventional cancer therapies.

The tumor suppressor gene TP53 (p53) is a central regulator of cellular stress responses and tumor suppression [11–13], mainly through its roles in cell cycle arrest [14], apoptosis [15], and other stress-adaptive processes. Recent studies have further revealed that p53 modulate ferroptosis in a context-dependent manner, at least in part by repressing SLC7A11 and thereby affecting cystine uptake and cellular antioxidant capacity [16–18]. Thus, p53 may serve as a potential molecular link between redox homeostasis and ferroptotic cell death in certain cancer contexts.

Recent pharmacological investigations have highlighted the therapeutic potential of natural products and bioactive compounds derived from traditional herbal medicines in breast cancer treatment [19, 20]. Among these, cephaeline (CPL), a spirocyclic alkaloid derived from traditional Asian medicinal systems [21, 22], has demonstrated empirical anti-tumor efficacy in head and neck squamous cell carcinoma models [23] and lung tumor [21]. Recent evidence suggests that cephaeline induces ferroptosis in lung cancer by disrupting NRF2-mediated antioxidant defense [24]. However, the molecular mechanisms underlying CPL’s anti-cancer effects in mammary tumor contexts remain poorly understood. In this study, we explored the therapeutic effect of CPL based on Ferroptosis on breast cancer, aiming to provide a new potential modality against breast tumor progression.

## Methods

### Reagents and cell culture

Cephaeline (CPL) was purchased from MCE Biotechnology (HY-N4118, Shanghai, China). According to the manufacturer’s information, CPL had a purity of ≥ 98%. CPL powder was stored at − 20 °C protected from light and was dissolved in dimethyl sulfoxide (DMSO) to prepare a stock solution. The stock solution was aliquoted and stored at − 20 °C to avoid repeated freeze–thaw cycles. For cell treatment, the CPL stock solution was diluted with complete culture medium to the indicated working concentrations, and the final DMSO concentration was kept below 0.1% in all groups, including the vehicle control. Murine 4T1 and human MDA-MB-231 cell lines were used as highly aggressive breast cancer models. 4T1 and MDA-MB-231 cancer Cells acquired from zqxzbio (Shanghai, China) were grown in RPMI-1640 medium enriched with 10% FBS (A5669701, Gibco, USA), 1% penicillin/streptomycin (G6784, Sigma-Aldrich, MO, USA), and incubated under 5% CO₂ and 37 °C with saturated humidity, and culture media were replaced every other day to support cell viability.

### Evaluation of cellular proliferation

The proliferative capacity of cells was measured using CCK-8 kit (BS350B, Biosharp, Hefei, China). In short [25], cells were plated into 96-well plates at a density of 2,000 cells per well and allowed to attach overnight under standard culture conditions. Cells were treated with CPL at concentrations ranging from 2.5 to 320 nM for 72 h to generate dose–response curves and determine IC50 values. Subsequently, 10 μL CCK-8 reagent was dispensed into each well, and the plates were maintained in a dark environment for 1 h. A microplate reader (BioRad, Richmond, CA, USA) was employed to assess absorbance at 450 nm. Cell viability was calculated relative to the untreated control group, and IC50 values were determined by nonlinear regression analysis using a four-parameter logistic dose–response model in GraphPad Prism software. The IC50 values were derived from three independent experiments and are presented with 95% confidence intervals. Based on the IC50 values determined from preliminary CCK-8 assays, representative concentrations of CPL corresponding to sub-IC50, near-IC50, and supra-IC50 levels were selected for subsequent experiments.

### Cell transfection

For RNA interference targeting TP53, 3 × 10^5^ cells were distributed into each well of a 6-well plate. Transfection was carried out using 20 μM TP53 siRNA (Ribio, Guangzhou, China) and Transfection kit following the manufacturer’s protocol.

### Reactive oxygen species (ROS) assay

ROS levels in CPL-treated 4T1 and MDA-MB-231 cells were assessed using dihydroethidium (DHE) (Sigma, MO, USA). The cells were exposed to 40 μM DHE for 1 h, rinsed with PBS, and the fluorescence signal was measured using a confocal microscope (Olympus FV3000, Tokyo, Japan). DHE fluorescence was detected using the red fluorescence channel at 594 nm, while DAPI nuclear staining was detected using the blue fluorescence channel. The relative DHE fluorescence intensity was quantified using ImageJ software.

### *Iron and Fe*^*2*+^*analysis*

After 72 h of CPL treatment, intracellular levels of ferric iron (Fe^3+^) and ferrous iron (Fe^2+^) were quantified using an iron levels were assessed using the Abcam (ab83366, MA, USA) iron detection kit, with procedures carried out per the recommended protocol.

### ELISA

Concentrations of MDA (RK09070) and GSH (RK04298) were measured using commercially available assay kits (Abclonal). Briefly, 2.5 × 10^4^ cells were distributed into each well of 96-well plates, followed by treatment with 80 nM CPL for 72 h. The liquid above the sediment was subsequently transferred to a fresh plate, and the reaction mixture was introduced following the manufacturer’s instructions. For each assay, standard curves were generated using serial dilutions of the provided standards, and MDA and GSH concentrations were calculated based on the corresponding standard curves. Absorbance was measured using a microplate reader (Thermo Fisher Scientific, MA, USA). All measurements were performed in triplicate, and each experiment was independently repeated at least three times.

### Western blot

RIPA lysis buffer enriched with protease inhibitors (Biosharp, Hefei, China) was used to extract proteins from cells [26]. Separation of proteins was carried out through SDS-PAGE, subsequently, protein samples were transferred to nitrocellulose membranes (Millipore, MA, USA), followed by blocking in 5% skim milk and incubation with specific primary antibodies at 4 °C overnight. Following extensive washing, the membranes were incubated with horseradish peroxidase (HRP)-conjugated secondary antibodies for 2 h at room temperature. Visualization of protein bands was accomplished using an ECL detection system. The primary antibodies utilized included GPX4 (1:1000, 52,455, CST, USA), SLC7A11 (1:1000, ab175186, Abcam), p53 (1:500, A19585, Abclonal), and GAPDH (1:5000, A19056, Abclonal). Band intensities were quantified using ImageJ software. The intensity of each target protein band was normalized to the corresponding GAPDH, and the relative protein expression level was calculated by comparison with the control group. Quantitative analysis was performed based on three independent experiments.

### qPCR

RNA extraction was performed with TRIzol (15,596,018, Invitrogen, USA). RNA pellets were resuspended in chloroform, and PrimeScript RT kit (Takara, Japan) was utilized to synthesize cDNA from the extracted RNA. PCR amplification was carried out using SYBR Green Master Mix (Takara, Japan). The 2-ΔΔCT approach was applied to determine relative gene expression, with 18S rRNA serving as the internal reference. Primer sequences were in Table 1.

**Table 1:** Target Gene

Target Gene	Forward(5′−3′)	Reverse(5′−3′)
*slc7a11*	TGAGAGCACGATGCATACACA	CCCTGCAGGTAACCTCCTTT
*18 s*	GTAACCCGTTGAACCCCATT	CCATCCAATCGGTAGTAGCG

### Cell scratch assay

Cells were seeded in a 6-well plate at a density of 2 × 10^5^ cells per well [27]. After confluence, a linear wound was generated using a 10 μL pipette tip to simulate cell migration. After scratching, the cells were cultured in low-serum medium containing 1% FBS for 24 h to minimize the influence of cell proliferation on wound closure while reducing the potential adverse effects of complete serum deprivation on cell viability and migration. Photographs of the initial wound and cells entering the scratched area were taken at 0 and 24 h using a light microscope (Olympus, Tokyo, Japan).

### Colony growth assay

A total of 200 cells per well were seeded into 6-well plates and maintained for 14 days, with the culture medium replaced every 3 days [28]. Colonies were fixed in 4% paraformaldehyde after three PBS rinses and then subjected to 1 h Giemsa staining. Residual dye was washed off and plates air-dried. Colonies with > 20 cells were counted, and relative colony-forming efficiency was adjusted and compared to control group.

### Statistical analysis

To ensure data reproducibility and reliability, all measurements were performed in triplicate, and each experiment was independently repeated at least three times. Statistical analyses were performed using GraphPad Prism software. Data are presented as mean ± standard deviation. For comparisons between two groups, an unpaired Student’s t-test was used. For comparisons among more than two groups, one-way analysis of variance (ANOVA) followed by Tukey’s multiple comparisons test was applied. For experiments involving multiple predefined comparisons, the corresponding reference groups are specified in the figure legends. A p value < 0.05 was considered statistically significant.

## Results

### CPL inhibits the proliferation and migration of breast cancer cells

**Fig. 1:**
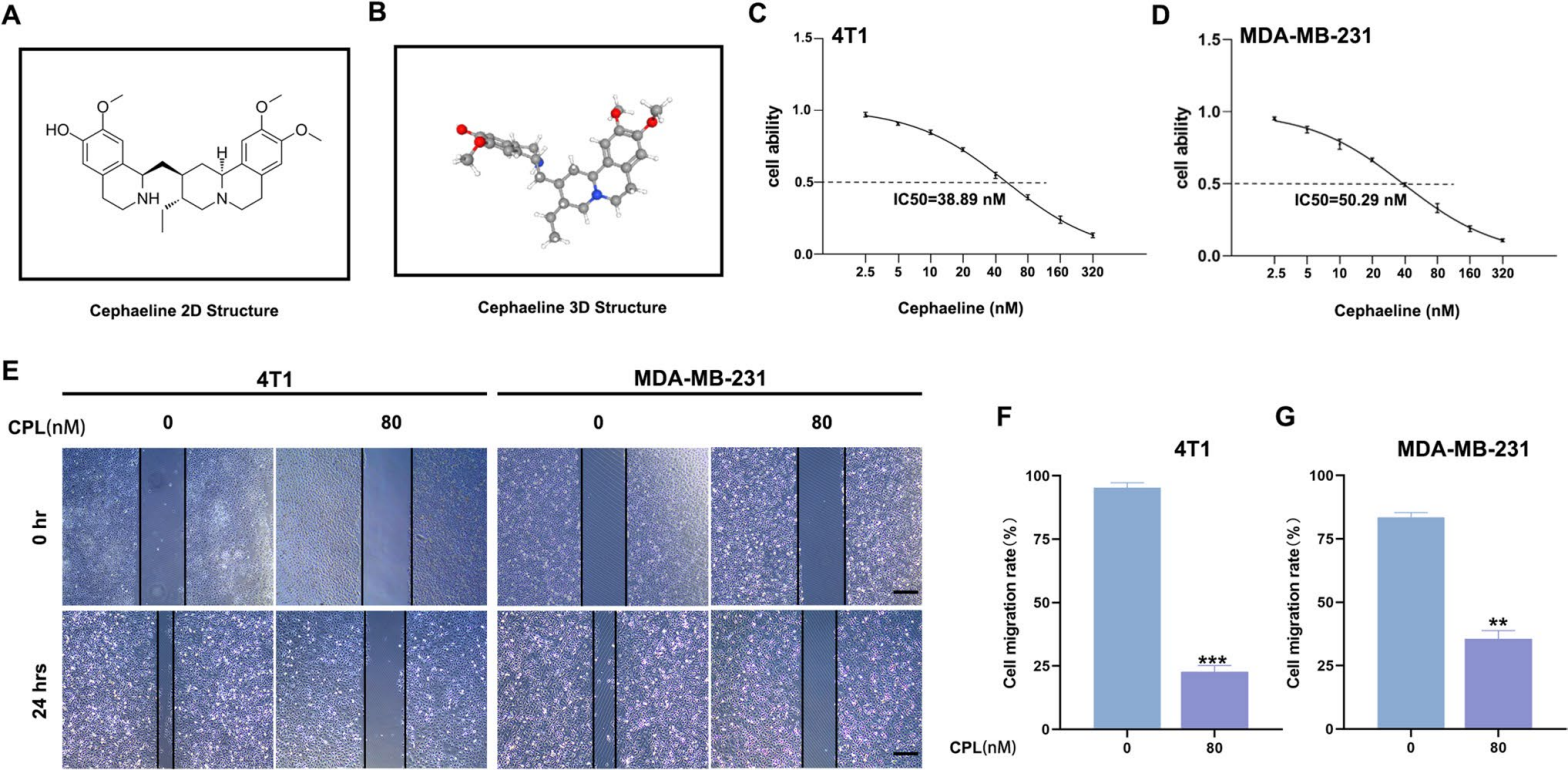
CPL Inhibits Breast Cancer Cell Proliferation and Migration. (**A-B**) Molecular structure of CPL. (**C-D**) IC50 values of CPL against 4T1 and MDA-MB-231 cells. (**E**) Scratch assay results showing the migration ability of 4T1 and MDA-MB-231 cells (Scale bar: 100 μm), (**F-G**) measured by ImageJ. All data are expressed as mean ± standard deviation (n = 3). Significance across groups was denoted by **p < 0.01, and ***p < 0.001

The chemical structure of CPL is illustrated in Fig. 1A-B. We initially assessed its impact on the proliferation of 4T1 and MDA-MB-231 cells, using CCK-8 assay. As depicted in Fig. 1C-D, CPL exhibited a dose-dependent inhibition of proliferation in both cell lines. The IC50 values, determined from the cell viability assay, were 38.89 nM (95% CI = 33.47–45.20 nM) for 4T1 and 50.29 nM (95% CI = 44.56–56.83 nM) for MDA-MB-231. Based on these IC50 values, three concentrations of CPL (20, 40, and 80 nM) were selected for subsequent experiments. Further evaluation using scratch assays revealed that CPL significantly impaired the migratory capacity of 4T1 (Fig. 1E-F) and MDA-MB-231 (Fig. 1E, G) cells.

### CPL treatment induces oxidative stress and ferroptosis in breast cancer cells

**Fig. 2:**
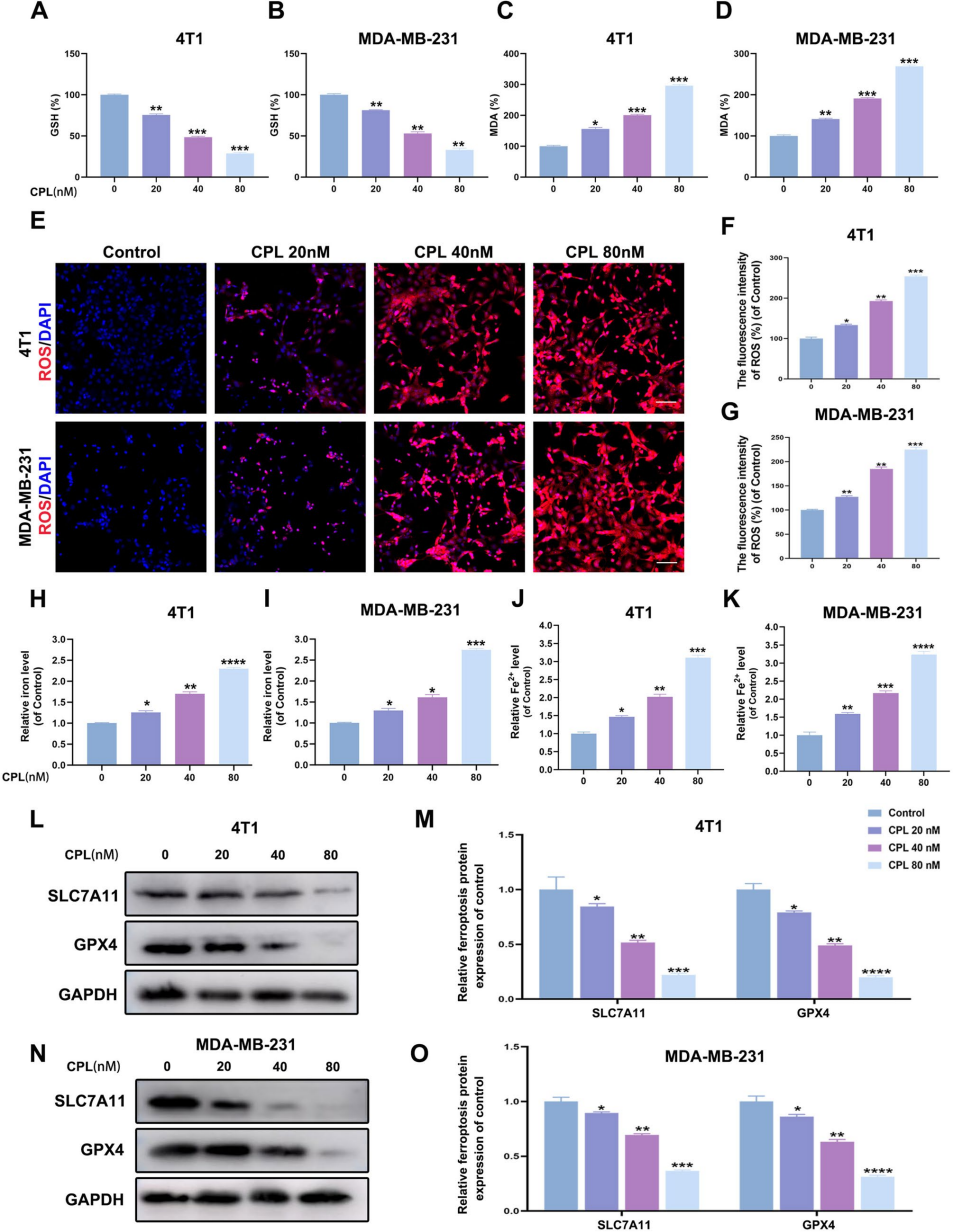
CPL Induces Ferroptosis in Breast Cancer Cells. (**A-D**) ELISA analysis of GSH and MDA levels in 4T1 and MDA-MB-231 cells treated with varying concentrations of CPL for 72 h. (**E**) Detection of ROS levels using the DHE assay kit (Scale bar: 50 μm), and (**F-G**) quantification of relative fluorescence intensity using ImageJ. (**H-I**) Measurement of total iron and (**J-K**) Fe.^2^⁺ levels using a commercial assay kit. (**L-O**) Western blot analysis of SLC7A11 and GPX4 protein expression in 4T1 and MDA-MB-231 cells following CPL treatment. All data are expressed as mean ± standard deviation (n = 3). Significance across groups was denoted by *p < 0.05, **p < 0.01, ***p < 0.001, and ****p < 0.0001

To elucidate the physiological mechanisms and therapeutic potential of CPL, we assessed the levels of GSH and MDA in CPL-treated cells. As illustrated in Fig. 2A-B, GSH levels exhibited a concentration-dependent decrease, while MDA levels showed a corresponding increase (Fig. 2C-D). Next, we assessed ROS levels after CPL treatment. Immunofluorescence analysis revealed a significant enhancement of cellular ROS fluorescence signals in cells, with the effect being concentration-dependent (Fig. 2E-G). Additionally, CPL treatment led to elevated levels of total iron and Fe^2+^ in cells (Fig. 2H-K). The expression of SLC7A11 and GPX4 was markedly downregulated following CPL treatment (Fig. 2L-O).

### CPL upregulates p53 expression in breast cancer cells

**Fig. 3:**
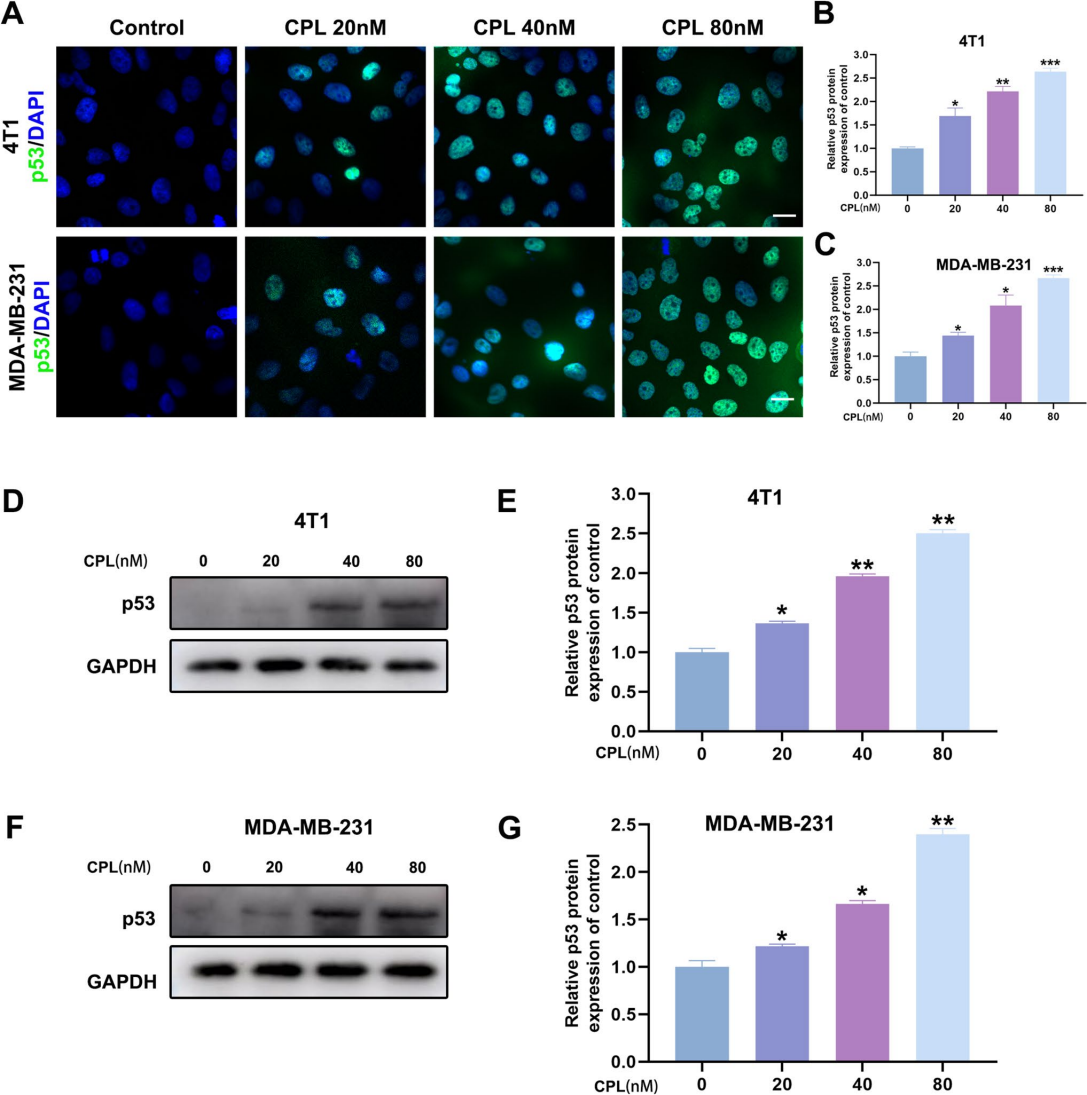
CPL Promotes p53 Expression. (**A**) Immunofluorescence staining of p53 (green) combined with DAPI nuclear counterstaining in cells, scale bar: 100 μm, and (**B-C**) quantification of relative fluorescence intensity using ImageJ. (**D-G**) p53 protein expression in 4T1 and MDA-MB-231 cells following CPL treatment. All data are expressed as mean ± standard deviation (n = 3). Significance across groups was denoted by *p < 0.05 and **p < 0.01

To further explore the mechanism underlying the effects of CPL, we examined whether CPL treatment affected p53 protein expression and subcellular localization in CPL-treated 4T1 and MDA-MB-231 cells. Immunofluorescence revealed a pronounced increase in nuclear p53 fluorescence intensity in both 4T1 and MDA-MB-231 cells following CPL treatment (Fig. 3A). suggesting enhanced nuclear accumulation of p53. Moreover, western blot analysis further confirmed that CPL increased p53 protein levels in a dose-dependent manner (Fig. 3B-E).

### Inhibition of p53 expression attenuates the ant-breast cancer activity of CPL

**Fig. 4:**
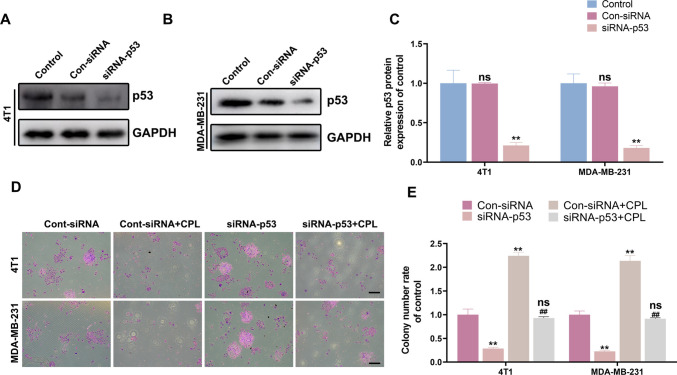
p53 Knockdown Abrogates CPL-Mediated Suppression of Breast Cancer. (**A-C**) Western blot analysis confirming the construction of the p53 silencing cell model. (**D-E**) Colony formation assay (Scale bar: 100 μm) evaluating the impact of p53 silencing and/or CPL treatment on colony-forming ability. All data are expressed as mean ± standard deviation (n = 3). Significance across groups was denoted by ns, not significant, **p < 0.01 and ***p < 0.001, ^##^p < 0.01 vs. Con-siRNA; ^###^p < 0.001 vs. siRNA-p53

Given the significant upregulation of p53 expression following CPL treatment, we investigated the role of p53 in mediating CPL's anticancer effects by silencing p53 expression in 4T1 and MDA-MB-231 using siRNA. Western blot confirmed the efficiency of p53 knockdown at the protein level (Fig. 4A-B). Functional assays revealed that the proliferation capacity of breast breast cancer cells was markedly elevated following p53 knockdown when compared with control siRNA-treated cells. In contrast, the proliferation capacity of cells in the Con-siRNA + CPL group was significantly diminished compared to the Con-siRNA group (Fig. 4C). Moreover, results from colony formation assays revealed that the inhibition of p53 substantially weakened CPL’s ability to suppress the colony-forming capacity in both cell lines (Fig. 4D-E).

### Silencing of the p53 gene blocks CPL-induced iron concentration and suppresses SLC7A11/GPX4 expression

**Fig. 5:**
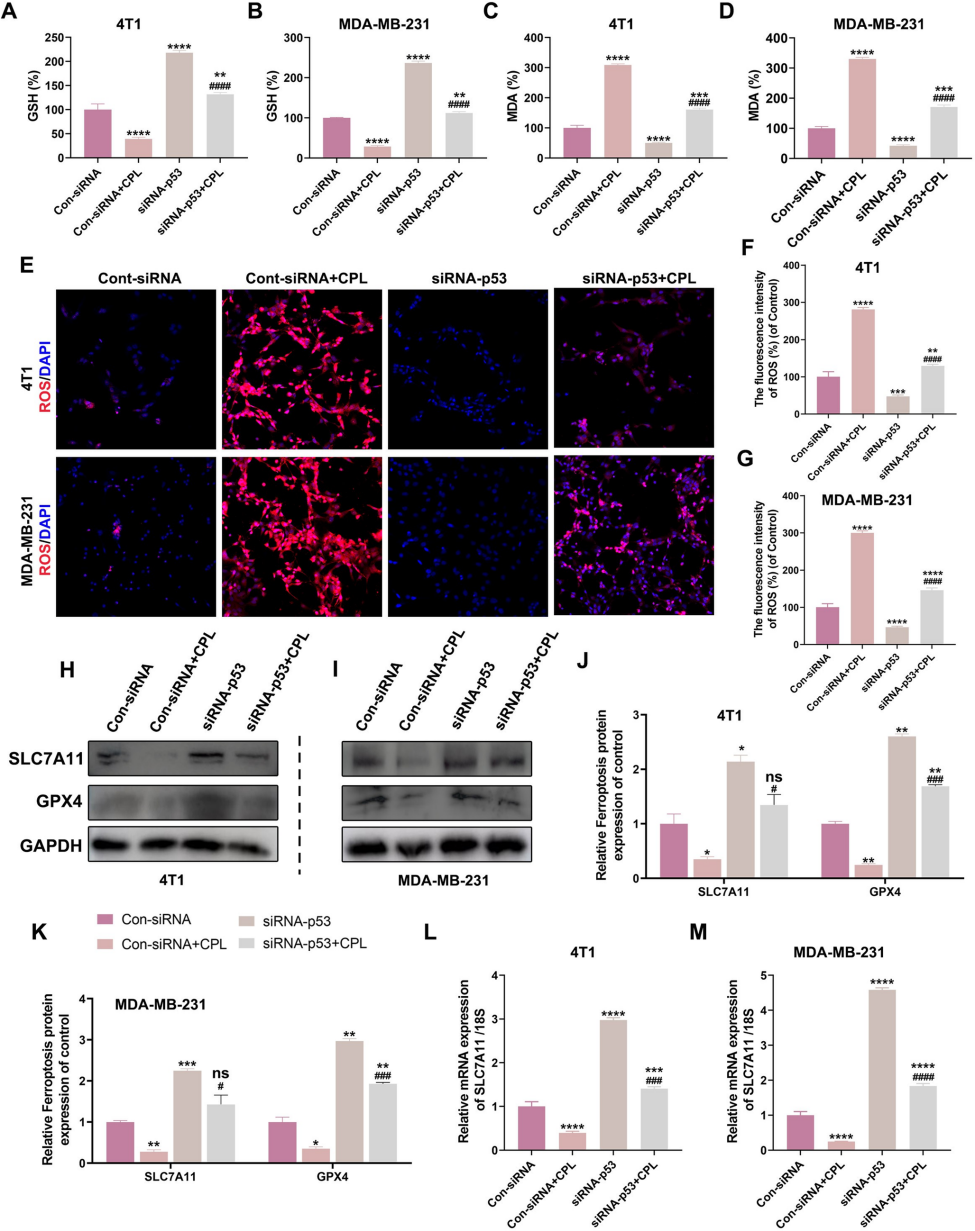
Silencing p53 Blocks CPL-Induced Ferroptosis. (**A-D**) Analysis of GSH and MDA levels following p53 knockdown and/or CPL exposure. (**E**) Detection of ROS levels using the DHE probe (scale bar: 100 μm), and (**F-G**) quantification of the relative mean fluorescence intensity (MFI) of DHE. (**H–K**) Western blot analysis of SLC7A11 and GPX4 protein expression, key indicators of ferroptosis. (L-M) qPCR to assess *slc7a11* levels. All data are expressed as mean ± standard deviation (n = 3). Significance across groups was denoted by **p < 0.01, ***p < 0.001, and ****p < 0.0001, ^###^p < 0.001 vs. Con-siRNA and.^####^p < 0.0001 vs. siRNA-p53

To investigate the role of p53 in CPL-induced iron accumulation and ferroptosis, siRNA was employed to knockdown the p53 gene in breast cancer cells. Following p53 knockdown, the CPL-induced reduction in intracellular GSH levels and elevation in MDA levels were significantly reversed (Fig. 5A-D). Similarly, the increase in intracellular ROS induced by CPL was markedly inhibited in the absence of p53 (Fig. 5E-G). We next examined whether p53 silencing could attenuate CPL-induced ferroptosis. Western blot results showed a pronounced decrease in SLC7A11 and GPX4 expression following CPL treatment in Con-siRNA–transfected cells, relative to untreated controls. Conversely, these levels were elevated in the siRNA-p53 group and showed a further increase in the siRNA-p53 + CPL group relative to the Con-siRNA + CPL group, although they did not reach the levels observed in the siRNA-p53 group alone (Fig. 5H-K). Additionally, p53 silencing reversed the CPL-induced downregulation of *slc7a11* expression (Fig. 5L-M).

## Discussion

Breast cancer remains a major global health challenge, and therapeutic resistance continues to limit the long-term efficacy of conventional treatments, including chemotherapy, radiotherapy, and targeted therapies [29–31]. Ferroptosis has emerged as an alternative therapeutic vulnerability, particularly in tumors resistant to apoptosis-based interventions [32]. Increasing evidence indicates that key ferroptosis regulators, such as GPX4 and SLC7A11, are closely associated with breast cancer progression, metastasis, and treatment resistance [33]. Notably, several clinically approved drugs, including sorafenib [34] and sulfasalazine [35], have been reported to exert antitumor effects at least in part through induction of ferroptosis via disruption of the SLC7A11-GPX4 antioxidant axis, highlighting the translational relevance of targeting ferroptosis-related pathways.

CPL has attracted attention for its antitumor activity in several malignancies [36]. Despite its emerging therapeutic promise, research elucidating its role and underlying mechanisms in breast cancer treatment remains limited. Our study demonstrates that CPL induces ferroptosis in breast cancer cells by activating the p53/SLC7A11/GPX4 axis. Mechanistically, CPL activates p53, leading to repression of SLC7A11, depletion of intracellular glutathione, downregulation of GPX4, and subsequent lipid peroxide and iron accumulation, ultimately triggering ferroptosis. Importantly, p53 knockdown markedly attenuated these effects, confirming its central regulatory role.

Despite these promising findings, several mechanistic and experimental limitations should be acknowledged. Although CPL treatment increased p53 protein accumulation and nuclear localization, the upstream events responsible for p53 regulation were not investigated. Potential mechanisms may involve MDM2-mediated p53 stabilization, ATM/ATR-related DNA damage responses, or ROS-associated stress signaling, which require further validation. In addition, NRF2 signaling should be considered, as previous studies have reported NRF2-related mechanisms in CPL-induced ferroptosis in lung cancer, and NRF2 can also regulate ferroptosis-associated genes such as SLC7A11 and GPX4. Thus, CPL-induced ferroptosis-related changes in breast cancer cells may involve broader redox-regulatory networks beyond p53 alone. Moreover, because p53 can regulate both apoptosis and ferroptosis, the potential contribution of apoptosis to CPL-induced cell death cannot be excluded. Future studies assessing NRF2 activity, upstream p53 regulatory events, and apoptosis-related markers will help clarify the relative contribution of these pathways.

From an experimental perspective, although multiple ferroptosis-related biochemical and molecular indicators were detected, ferroptosis inhibitor-based rescue experiments using Ferrostatin-1, DFO, or Liproxstatin-1 were not performed. Established ferroptosis inducers, such as Erastin or RSL3, were also not included as positive controls. These experiments would provide more direct evidence for ferroptosis dependence and help contextualize the relative potency of CPL. In addition, Transwell migration and invasion assays, mitochondrial morphology analysis, and broader validation in subtype-defined breast cancer cell panels were needed in future research. Since only 4T1 and MDA-MB-231 cells were used, both representing aggressive triple-negative-like models, future studies should include hormone receptor-positive models such as MCF-7, HER2-positive breast cancer cells to determine whether CPL sensitivity differs across breast cancer subtypes. Finally, this study was conducted entirely in cell culture models, future in vivo studies are needed to evaluate its antitumor efficacy, ferroptosis-related effects in tumor tissues, pharmacokinetic properties, systemic toxicity, and therapeutic window. In addition, the potential synergy or antagonism between CPL and existing anticancer therapies warrants further investigation.

### Conclusion

In this study, we investigated the anticancer effects of CPL in breast cancer cells and demonstrated that CPL induces ferroptosis through activation of the p53/SLC7A11/GPX4 axis. These findings elucidate a previously unrecognized mechanism underlying the antitumor activity of CPL and highlight its role as a modulator of ferroptosis-related redox homeostasis.

## Data Availability

No datasets were generated or analysed during the current study.
